# Genetic diversity of the Yokose virus, XYBX1332, isolated from bats (*Myotis daubentonii*) in China

**DOI:** 10.1186/s12985-018-1107-3

**Published:** 2019-01-11

**Authors:** Yun Feng, Xiaojie Ren, Ziqian Xu, Shihong Fu, Xiaolong Li, Hailin Zhang, Weihong Yang, Yuzhen Zhang, Guodong Liang

**Affiliations:** 1 Yunnan Provincial Key Laboratory for Zoonosis Control and Prevention, Yunnan Institute of Endemic Diseases Control and Prevention, Dali, China; 2 State Key Laboratory of Infectious Disease Prevention and Control, National Institute for Viral Disease Control and Prevention, Chinese Center for Disease Control and Prevention, Beijing, China; 3 Collaborative Innovation Center for Diagnosis and Treatment of Infectious Diseases, Hangzhou, China; 4 Department of Anesthesiology, The First Affiliated Hospital of Guangzhou University of Chinese Medicine, Guangzhou, China

**Keywords:** Yokose virus, Bat, China

## Abstract

**Background:**

Yokose virus was first isolated from bats (*Miniopterus fuliginosus*) collected in Yokosuka, Japan, in 1971, and is a new member of the family *Flaviviridae*, genus *Flavivirus*. In this study, we isolated a Yokose virus from a serum sample of *Myotis daubentonii* (order Chiroptera, family Vespertilionidae) collected in Yunnan province, China in 2013.

**Methods:**

The serum specimens of bat were used to inoculate in BHK-21 and Vero E6 cells for virus isolation. Then the viral complete genome sequence was obtained and was used for phylogenetic analysis performed by BEAST software package.

**Results:**

The virus was shown to have cytopathic effects in mammalian cells (BHK-21 and Vero E6). Genome sequencing indicated that it has a single open reading frame (ORF), with a genome of 10,785 nucleotides in total. Phylogenetic analysis of the viral genome suggests that XYBX1332 is a Yokose virus (YOKV) of the genus *Flavivirus*. Nucleotide and amino acid homology levels of the ORF of XYBX1332 and Oita-36, the original strain of YOKV, were 72 and 82%, respectively. The ORFs of XYBX1332 and Oita-36 encode 3422 and 3425 amino acids, respectively. In addition, the non-coding regions (5′- and 3′-untranslated regions [UTRs]) of these two strains differ in length and the homology of the 5′- and 3′-UTRs was 81.5 and 78.3%, respectively.

**Conclusion:**

The isolation of YOKV (XYBX1332) from inland China thousands of kilometers from Yokosuka, Japan, suggests that the geographical distribution of YOKV is not limited to the islands of Japan and that it can also exist in the inland areas of Asia. However, there are large differences between the Chinese and Japanese YOKV strains in viral genome.

## Background

The *Flavivirus* includes 70 members [1], etc., many of which, including Japanese encephalitis virus [2], dengue virus [3], West Nile virus [3], yellow fever virus [4], and Zika virus [5], which can cause some human severe diseases [6]. Flaviviruses have introduced a substantial disease burden in humans and animals around the world, and, as mosquito-borne viruses, can spread widely [6, 7].

**Fig. 1:**
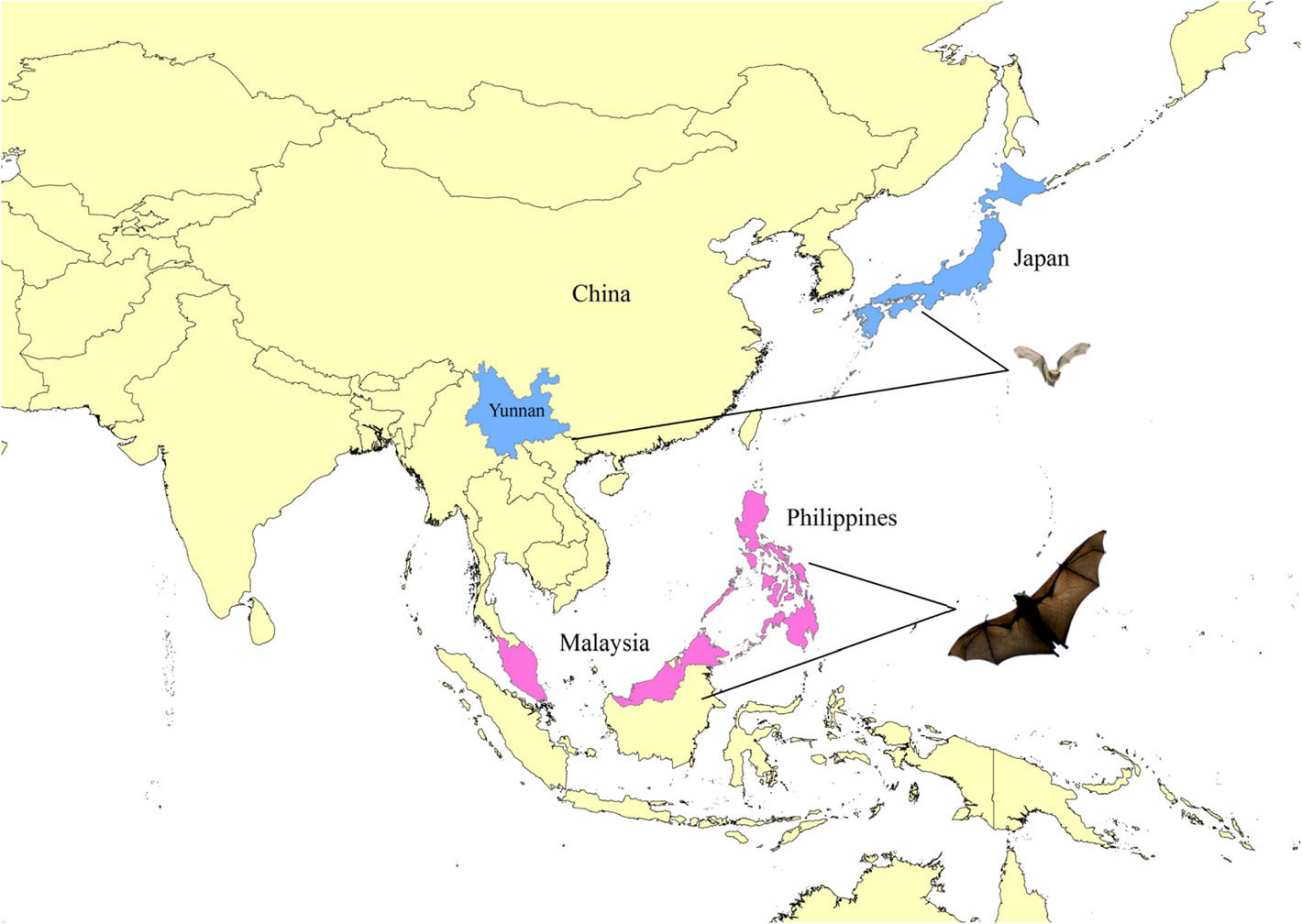
Isolation of Yokose virus (YOKV) and distribution of areas positive for the YOKV antibody. YOKV was isolated from microbats in Japan and China (the blue areas), and YOKV antibodies were detected in the serum of fruit bats in Malaysia and Philippines (the pink areas)

Yokose virus (YOKV) belongs to the *Flavivirus*, which was first isolated from bats (*Miniopterus fuliginosus*) collected in Yokosuka, Japan, in 1971, and the first YOKV strain isolated was Oita-36 (Fig. 1). Sequencing results indicate that the YOKV genome is 10,857 nucleotides (nt) in total [8]. Like other flaviviruses, YOKV has only one open reading frame (ORF), which encodes 3425 amino acids. The genome encodes three structural proteins, the capsid (C), membrane (M), and envelope (E) proteins, as well as seven non-structural proteins (NS1, NS2A, NS2B, NS3, NS4A, NS4B, and NS5). There are 5′ and 3′ non-coding regions at the ends of the genome. Molecular genetic analysis indicated that YOKV is a new member of the family *Flaviviridae*, genus *Flavivirus* [8]. Furthermore, YOKV is genetically related to other flaviviruses, including Japanese encephalitis virus, dengue virus, yellow fever virus, and West Nile virus. YOKV is closely related to yellow fever virus, which both clustered in the same clade [8]. Additionally, YOKV was neutralized by antisera to individuals inoculated with yellow fever virus vaccine. The above results suggested that this new member of flavivirus is genetically related to yellow fever virus. A serological survey of YOKV in the Philippines and Malaysia showed that the YOKV antibody was detected in the serum of bats (*Rousettus leschenaultii*), suggesting that the distribution of YOKV was not limited to Yokosuka Island, Japan [9] (Fig. 1).

In this study, we isolated a virus (XYBX1332) from a serum sample of *Myotis daubentonii* (order Chiroptera, family Vespertilionidae) collected in the Yunnan-Guizhou Plateau Region (average altitude 2000–4000 m) in the southwest of China in July 2013. XYBX1332 was identified as YOKV using whole-genome sequencing. However, our study indicated some clear differences in molecular biology between XYBX1332 and the original isolate of YOKV.

## Methods

### Sample collection

The bat specimens were collected in Xiangyun County of Dali Prefecture, Yunnan Province (100.32°E, 25.28°N). Sticky nets were placed at the outlet of the cave in the evening. Once trapped, the bats were classified according to the morphological characteristics. The bats were released after the blood samples were collected. The blood samples were kept in − 80°[10].

### Cell lines and virus isolation

Baby hamster kidney (BHK-21) cells were stored in the laboratory. The cells were cultured in minimal essential medium (MEM; Gibco, Grand Island, NY, USA) containing 10% fetal bovine serum (FBS; Invitrogen, Carlsbad, CA, USA), 1% penicillin-streptomycin combination, 1% glutamine, and 2% NaHCO_3_ (pH 7.4) at 37 °C in a 5% CO_2_ atmosphere. African green monkey kidney cells (Vero E6) were cultured in a similar manner [10–12].

The serum specimens of bat were diluted 1:50 and inoculated in monolayered BHK-21 and Vero E6 cells, followed by incubation at 37 °C in a 5% CO_2_ atmosphere. Cells were observed daily for cytopathic effects (CPEs). [10, 11].

### Viral RNA extraction

Viral RNA was extracted from 140 μl of culture supernatant of XYBX1332 cultured in BHK-21 cells using a QIAamp Viral RNA Extraction Kit (Qiagen, Hilden, Germany). Then, cDNA was generated by reverse transcription using Ready-to-Go™ You Prime First-Strand Beads (GE Healthcare, Little Chalfont, UK). Total RNA was extracted from the culture supernatant of BHK-21 cells infected with XYBX1332 and a cDNA library was generated by reverse transcription. Then, primers specific for *Alphavirus*, *Flavivirus*, and *Bunyavirus* were used to perform polymerase chain reaction (PCR) amplification (Table 1). PCR products were detected by 1% agarose gel electrophoresis [13–15].

**Table 1:** Arbovirus-specific primers used for amplification in this study

	Sequence of primers(5′-3′)	Amplify region	Length of product	References
*Flavivirus*				
FU1	TACCACATGATGGGAAAGAGAGAGAA	NS5	310	13
CFD2	GTGTCCCAGCCGGCGGTGTCATCAGC			
*Alphavirus*				
M2W	YAGAGCDTTTTCGCAYSTRGCHW	NS1	434/310	13
cM3 W	ACATRAANKGNGTNGTRTCRAANCCDAYCC			
M2 W2	TGYCCNVTGMDNWSYVCNGARGAYCC			
*Bunyavirus*				
BUP	ATGACTGAGTTGGAGTTTGATGTCGC	S	251	13, 14
BDW	TGTTCCTGTTGCCAGGAAAAT			

### Viral genome sequencing

The target gene fragment of XYBX1332 was amplified using the designed primers (Primer 5.0 software; Table 2). Amplified products were examined by agarose gel electrophoresis (1%), purified using a QIAquick Gel Extraction Kit (Qiagen), and sequenced directly [10, 13, 14].

**Table 2:** Primers designed for XYBX1332

Primers	Sequences (5′ - 3′)	Sites
Yok-F1	5’ TTTGCGTGCTAGTCGCTGAG 3’	9–37
Yok-R1	5’ TATCCTTTGCCGTAAGAGTGA 3’	1119–1139
Yok-F2	5’ CCCTGCATACAGCACTCATT 3’	995–1014
Yok-R2	5’ GCCTTTCATTGTCAGTCCCT 3’	1880–1898
Yok-F3	5’ GGCTGGAGCTACTAGAATTACG 3’	1781–1803
Yok-R3	5’ TCACTGATGCTATTTCCCTTG 3’	2613–2633
Yok-F4	5’ AGGCGTGAAATCAAGTGT 3’	2505–2422
Yok-R4	5’ CATACCAGCATTCATT 3’	3459–3474
Yok-F5	5’ CAGTAAGAGGGGACCATCAGT 3’	3353–3573
Yok-R5	5’ ATAGCACAGCAATAGCACAGAA 3’	4356–4377
Yok-F6	5’ TGGAATGACGGTGATAGGAG 3’	4238–4257
Yok-R6	5’ GGCAATGGCTGAAACAAAT 3’	5120–5138
Yok-F7	5’ ATAGTCAACAAACAAGGGGAAGT 3’	5037–5059
Yok-R7	5’ CAGAGGAAGCAGTTATTGGAAGT 3’	5976–5998
Yok-F8	5’ TACCCGTGGAAAGAGTGATAGA 3’	5869–5890
Yok-R8	5’ GCATAGAATAAAGAAGACAAGCATA 3’	6815–6839
Yok-F9	5’ CCAGGATGACATTGGCTTTT 3’	6703–6720
Yok-R9	5’ CAGCAGCACCGTCTTGGA 3’	7819–7836
Yok-F10	5′ AGGTGAGATGTGGAAGAAGGA 3’	7700–7720
Yok-R10	5’ AGGAGCGGTATGGGTGG 3’	8586–8602
Yok-F11	5’ GATTCAGGGACCAGAAGTGTT 3’	8454–8474
Yok-R11	5’ CTCCTTTCAATGGTCTTTCAACTCT 3’	9438–9462
Yok-F12	5’ TCTTGGCTGAAGCGGTAAT 3’	9367–9385
Yok-R12	5’ CAGACTCTATTCCATACATCAAGC 3’	10,135–10,158
Yok-F13	5’ CGATCAATTCTTCAGTGCCAATA 3’	10,018–10,040
Yok-R13	5’ CGTCCAAACAAGAGGAAAAT 3’	10,526–10,545

### Sequence amplification of non-coding regions

The 5′ and 3′-UTR sequences were amplified using RACE System Rapid Amplification of cDNA Ends Kit (Invitrogen), in accordance with the manufacturer’s instructions. The sequences obtained were used to assemble a complete genome sequence.

### Phylogenetic analysis

Sequences of typical strains of *Flavivirus* isolated from different hosts in various years and countries were downloaded from GenBank (Table 3). The SeqMan in DNASTAR was used for sequence assembly and quality analysis. The homology analysis and alignment of nucleotide and amino acid sequences were conducted using BioEdit (version 7.0.5.3) and ClustalX 1.8 software. Analysis of differences in the nucleotide and amino acid sequences was conducted using GeneDOC software [10, 13, 14].

**Table 3:** Sequences used for phylogenetic analysis in this study

Virus	Strain	Year	Country	Source	Accession No.
Japanese encephalitis virus (JEV)	Ishikawa	1994	Japan	Swine	AB051292
Japanese encephalitis virus (JEV)	FU	1995	Australia	Human serum	AF217620
Japanese encephalitis virus (JEV)	p3	1949	China	Human brain	U47032
Japanese encephalitis virus (JEV)	JKT6468	1981	Indonesia	Culex tritaeniorhynchus	AY184212
Japanese encephalitis virus (JEV)	Muar	1952	Malaysia	Human brain	HM596272
Murray Valley encephalitis virus (MVEV)	MVE-1-51	1951	Australia	Human brain	NC_000943
West Nile virus (WNV)	ArB3573/82		Central African Republic		DQ318020
Kunjin virus (KUNV)	MRM61C		Australia	Mosquito	D00246
St. Louis encephalitis virus (SLEV)	Kern217		USA	Mosquito	DQ525916
Zika virus	MR766-NIID	1947	Uganda	Monkey	LC002520
Zika virus	ZikaSPH2015	2015	Brazil	Human	KU321639
Dengue virus 4 (DENV4)	341,750	1982	Colombia	Human	GU289913
Dengue virus 2 (DENV2)	D2/SG/05K4155DK1/2005	2005	Singapore	Human blood	EU081180
Dengue virus 1 (DENV1)	SG(EHI)D1227Y03	2003	Singapore		FJ469909
Dengue virus 3 (DENV3)	D3/H/IMTSSA-MART/1999/1243	1999	Martinique (French West Indies)	Human blood	AY099337
Yellow fever virus (YFV)	YFV17D				X03700
Yokose virus(YOKV)	XYBX1332	2013	China	Bat	–
Yokose virus(YOKV)	Oita-36	1971	Japan	Bat	AB114858
Powassan virus (POWV)	Spassk-9	1975	Russia	Dermacentor silvarum (tick)	EU770575
Langat virus (LANV)	TP21			Tick	NC_003690
Louping ill virus (LIV)	369/T2		UK		NC_001809
Tick-borne encephalitis virus (TBEV)	Toro-2003	2003	Sweden: Toro	Ixodes ricinu	DQ401140
Culex flavivirus	Tokyo	2003	Japan	*Culex pipiens*	AB262759

Nucleotide sequence phylogenetic trees were reconstructed using Markov chain Monte Carlo analysis implemented in the BEAST software package (version 1.8.4; http://beast.bio.ed.ac.uk/). The analysis was performed using the generalized time reversible substitution model with gamma distributed rate variation among sites and using default priors. The Markov chains were run for 10 million generations, with the first 10% of samples discarded as burn-in. Convergence of parameters was verified using Tracer v1.4 and indicated as effective sample size (ESS > 200). A maximum clade credibility tree was summarized using TreeAnnotator, which annotates all nodes with posterior probability support values [16].

## Results

### Virus isolation

**Fig. 2:**
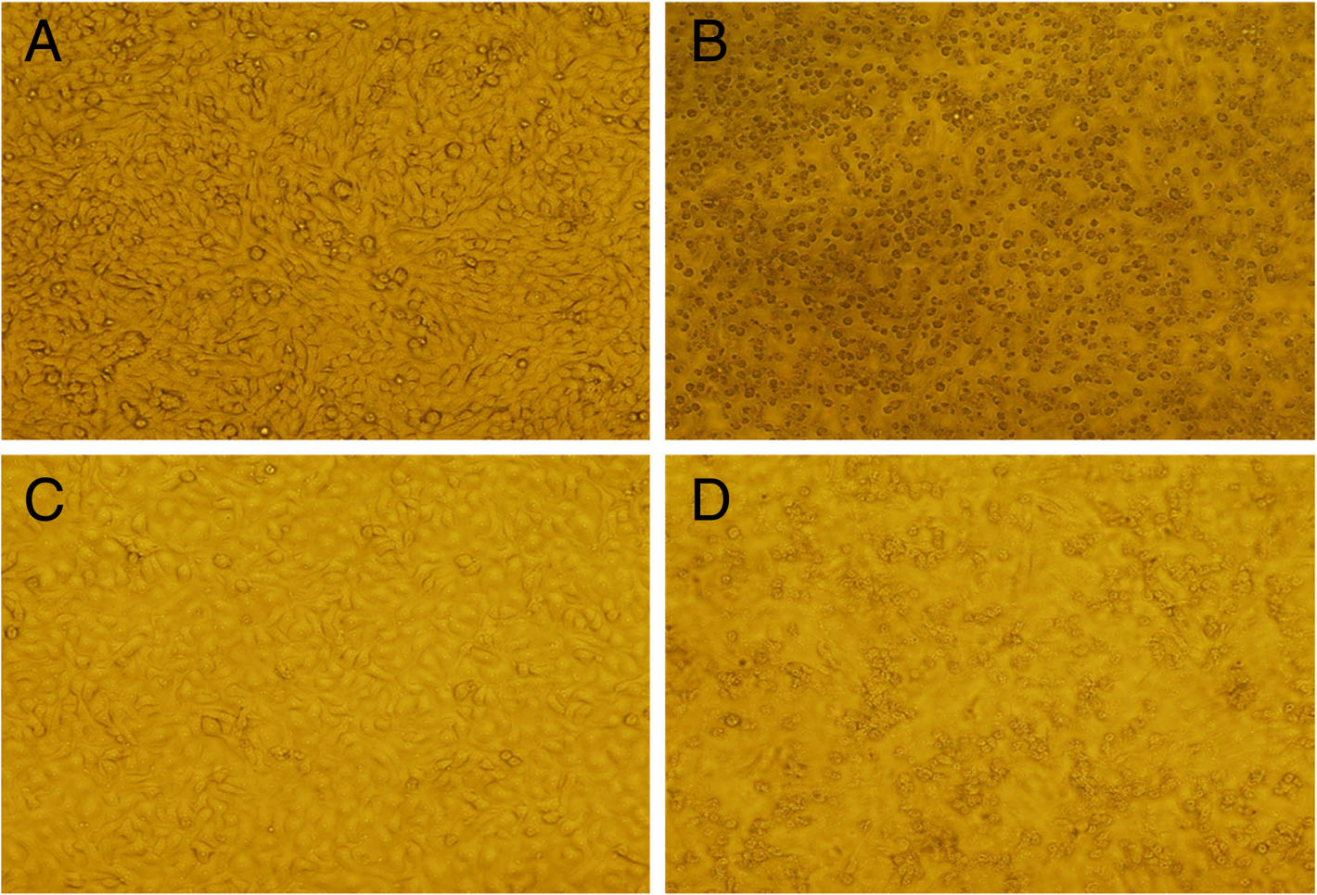
Cytopathic effects (CPEs) of XYBC1332 in BHK-21 and Vero E6 cells. **a**) Control uninfected BHK-21 cells. **b**) Infected BHK-21 cells at 4 days post-infection. **c**) Control uninfected Vero E6 cells. **d**) Infected Vero E6 cells at 5 days post-infection

Both BHK-21 and Vero E6 cells inoculated with serum specimen numbered XYBX1332 exhibited obvious CPEs, which included rounding up, aggregation, and exfoliation (Fig. 2).

### Virus identification

The results showed that PCR products were detected with *Flavivirus*-specific primers, while no amplification was detected with primers specific for *Alphavirus* and *Bunyavirus*, suggesting that XYBX1332 is a *Flavivirus*.

### Viral genome sequencing and homology analysis

To determine the molecular characteristics of XYBX1332, the whole genome of XYBX1332 was amplified using 13 pairs of primers (Table 2) and sequenced (GenBank accession no. MH051229).

The Open Reading Frame (ORF) of XYBX1332 is 10,266 nt (excluding the 5′ and 3′-UTRs), which encodes 3422 amino acids and 10 protein-coding genes. Nucleotide and amino acid sequence information is provided in Table 4.

**Table 4:** Open Reading Frame (ORF) Genome homology of the XYBX1332, Oita-36 and YFV17D

Protein	XYBX1332		Oita-36				YFV17D			
	nt^a^	aa^b^	%^c^	nt^d^	%^e^	aa^f^	% ^g^	nt^h^	% ^i^	aa^j^
C	378	126	71	384	69	128	49	363	34	121
PrM	504	168	71	504	79	168	44	492	47	164
E	1470	490	74	1470	83	490	57	1479	49	493
NS1	1059	353	76	1059	86	353	57	1056	53	352
NS2A	684	228	60	681	66	227	46	672	26	224
NS2B	390	130	54	390	79	130	26	390	32	130
NS3	1860	620	73	1860	85	620	59	1869	54	623
NS4A	447	149	72	447	83	149	47	447	36	149
NS4B	756	252	71	762	83	254	53	750	44	250
NS5	2718	906	74	2718	85	906	61	2715	62	905
ORF	10,266	3422	72	10,275	82	3425	54	10,233	51	3411

XYBX1332 is the strain isolated in this study. Oita-36 and YFV17D are the reference viruses. GENETYX Ver.11 software was used in the comparison of nucleotide and amino acid sequence length in the region. ^a^ and ^b^ represent the nucleotide sequence length (nt.) and amino acid sequence length (aa.) of XYBX1332 respectively; ^d^, ^f^ represents Oita-36 length of nt. and aa. respectively; ^h^, ^j^ represents YFV17D length of nt. and aa. respectively. ^c^ and ^e^ represent homology of nucleotide and amino acid between XYBX1332 and Otia-36. ^g^ and ^i^ represent homology of nucleotide and amino acid between XYBX1332 and YFV17D

Compared with other flaviviruses, YOKV shared the highest homology with yellow fever virus [8]. Therefore, we determined the nucleotide and amino acid homology of YOKV (Oita-36 strain), yellow fever virus (YFV17D strain) and XYBX1332. The analysis showed that the nucleotide homology of XYBX1332 and Oita-36 ranged from 54 to 76%, meanwhile the amino acid homology ranged from 66 to 86%. The nucleotide and amino acid homology levels of the coding region between these two viruses were 72 and 82%, respectively. The nucleotide and amino acid homology levels of XYBX1332 with YFV17D were in the ranges of 26–63% and 26–62%, respectively. The nucleotide and amino acid homology levels of the coding regions of XYBX1332 and YFV17D were 54 and 51%, respectively. Further analysis revealed differences in the number of amino acids encoded by XYBX1332 and Oita-36 in the C gene (XYBX1332/Oita-36: 126/128), NS2A gene (228/227), and NS4B gene (252/254). The homology of XYBX1332 and Oita-36 is shown in Table 4.

### Phylogenetic analysis

**Fig. 3:**
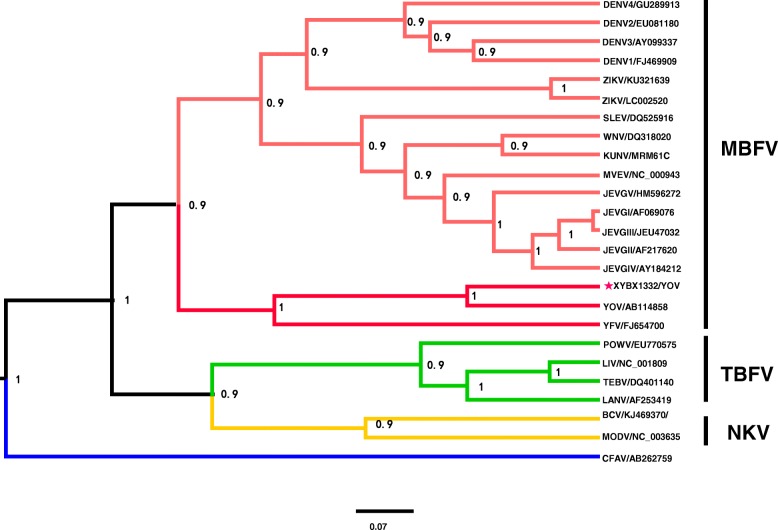
Bayesian phylogeny of the genus *Flavivirus* based on open reading frame gene nucleotide sequence. Posterior probability values of each cluster are shown to the right of the nodes. The mosquito-borne, tick-borne, and no known-vector flaviviruses (MBFV, TBFV, and NKV, respectively) are labeled in red, green, and yellow, respectively. The newly isolated virus in our study is indicated with a red star. Scale bars indicate the number of nucleotide substitutions per site

To understand the molecular genetic evolution of XYBX1332, a phylogenetic analysis was conducted based on the ORF sequences of XYBX1332 and 23 other strains of flavivirus (including mosquito- and tick-borne viruses; Table 3). The results suggested that these strains could be divided into three evolutionary branches. The first branch contained 18 strains of mosquito-borne flavivirus, including Japanese encephalitis virus, West Nile virus, Dengue virus, and Zika virus, while four tick-borne virus strains, including tick-borne encephalitis virus and Powassan virus, were grouped in the second branch. The third branch incorporated only one strain, a Culex flavivirus isolated in Tokyo in 2003 (Fig. 3). XYBX1332 isolated from bats in this study shared the same evolutionary branch as YOKV (Oita-36) isolated in Yokosuka, Japan, along with other mosquito-borne viruses, including Japanese encephalitis virus.

### 5′- and 3′-UTR sequences

**Fig. 4:**
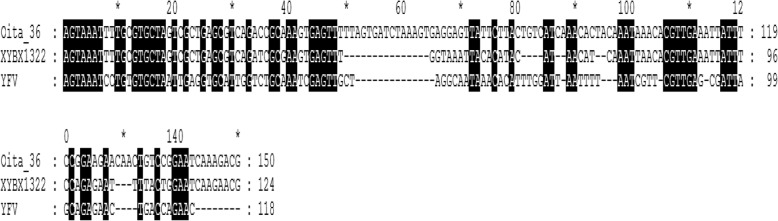
5′-UTR sequence of XYBX1332

**Fig. 5:**
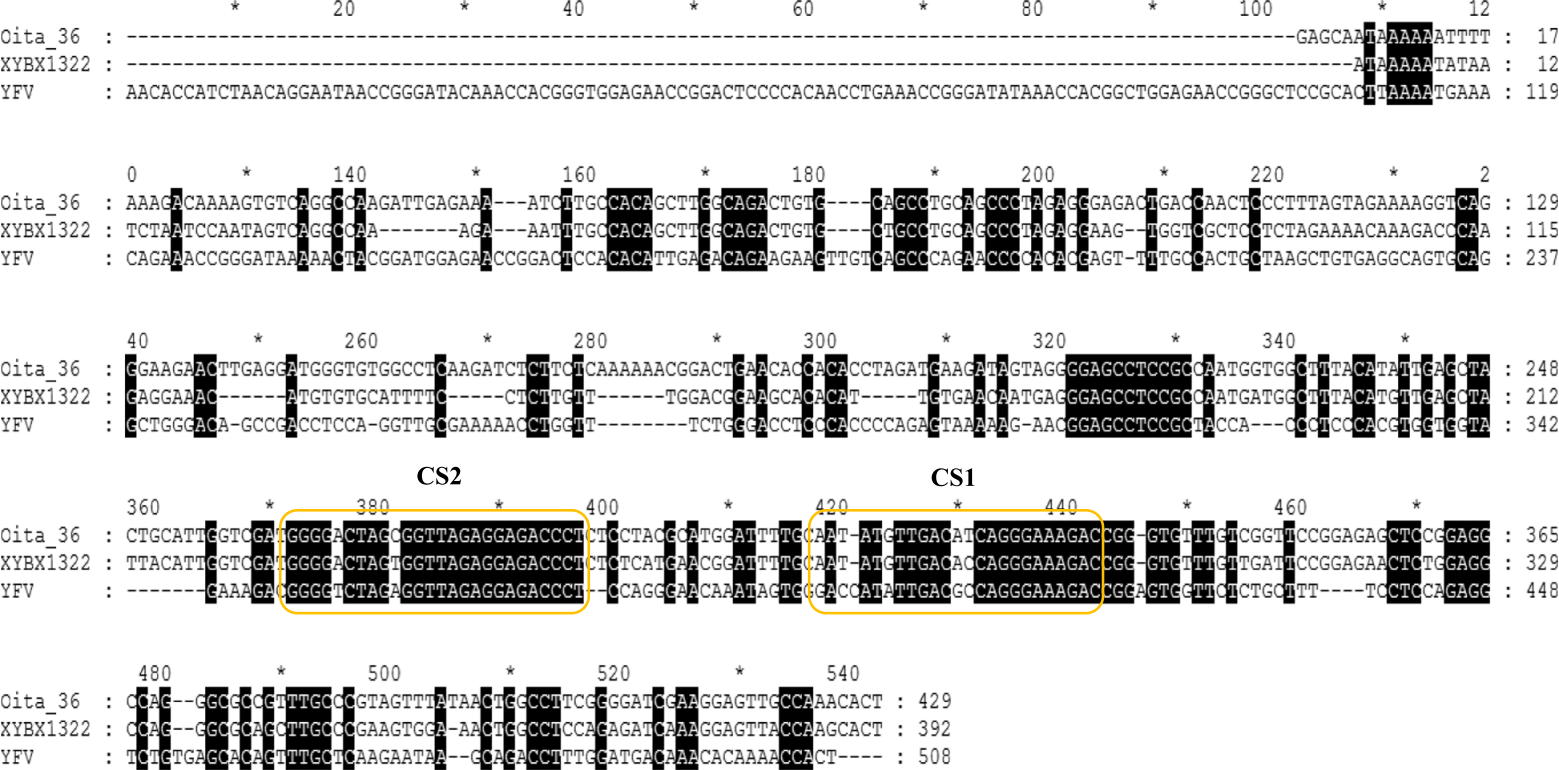
3′-UTR sequence of XYBX1332. The yellow boxes indicate the two conserved motifs, CS1 and CS2

The 5′ and 3′-UTR sequences were amplified using RACE System Rapid Amplification of cDNA Ends Kit. Sequencing of the non-coding region of XYBX1332 showed that the lengths of its 5′- and 3′-UTR sequences are 124 and 395 nt, respectively. Furthermore, the length of the 5′-UTR of XYBX1332 is 26 nt shorter than that of Oita-36 (23 and 3 base deletions at loci 63–86 and 122–123, respectively); while the 3′-UTR sequence is 37 nt shorter than that of Oita-36 (12, 19, and 5 base deletions at loci 10,444–10,460, 10,574–10,592, and 10,595–10,599, respectively). The 5′-UTR sequence of XYBX1332 exhibited 81.5 and 35.6% homology with Oita-36 and YFV17D, and there was 78.3 and 16.6% homology for the 3’-UTR sequence, respectively (Table 5 and Fig. 4). Additionally, compared with Oita-36, one base mutation (C > T) occurred in CS2, one of two conserved motifs in the 3′-UTR sequence of XYBX1332 (Fig. 5).

**Table 5:** 5′- and 3′-untranslated region (UTR) Genome homology of the XYBX1332, Oita-36 and YFV17D

	XYBX1322	Oita-36		YFV17D	
	nt^a^	nt^b^	%^d^	nt^c^	%^e^
5’-UTR	124	150	81.5	118	35.6
3’-UTR	395	432	78.3	508	16.6

XYBX1332 is the strain isolated in this study. Oita-36 and YFV17D are the reference viruses. Percent identity value calculated based on alignment. ^a^, ^b^ and ^c^ represent the nucleotide sequence length (nt.) of XYBX1332, Otia-36 and YFV17D respectively. ^d^ represents homology of nucleotide between XYBX1332 and Otia-36. ^e^ represents homology of nucleotide between XYBX1332 and YFV17D

## Discussion

In this study, the virus XYBX1332 was isolated from bat specimens collected in Yunnan Province, mainland China. This virus was identified as a YOKV in the genus *Flavivirus* based on phylogenetic analysis (Fig. 3). However, some clear differences between XYBX1332 and the original strain of YOKV (Oita-36) were identified as following.1) As mentioned above, the genome length of Oita-36 (including the 5′- and 3′-UTRs) is 10,857 nt, while that of XYBX1332 is 10,785 nt. 2) The coding region of Oita-36 is 10,275 nt, which encodes 3425 amino acids; The coding region of XYBX1332 is 10,266 nt, which encodes 3422 amino acids (Table 4). 3) The two viruses of ORF region exhibited 72 and 82% nucleotide and amino acid homology, respectively.4) The lengths of the 5′-UTR for XYBX1332 and Oita-36 are 124 and 150 nt, respectively, while those of the 3′-UTR are 395 and 432 nt, respectively. The homology of the 5′- and 3′-UTR sequences between the two strains was determined to be 81.5 and 78.3%, respectively (Table 5).5) It is only 74% of the nucleotide homology in the 3’end of NS5 gene between XYBX1332 virus and Oita-36 virus (Table 4), which is much lower than cut-off (84%) of that between species of *Flavivirus*[17]. These results suggested that, although XYBX1332 is a YOKV based on phylogenetic analysis, it may be a new virus in *Flavivirus*. Furthermore, the cross-neutralization test between XYBX1332 virus and Oita-36 virus would be conducted by the antigenicity between the viruses to confirm whether the XYBX1332 virus is a new species in *Flavivirus*.

As mentioned earlier, YOKV (Oita-36) was first isolated from a bat (*M. fuliginosus*) in Japan, while XYBX1332 was isolated from the serum of *M. daubentonii*. Although the strains were isolated from different bat species, *Miniopterus fuliginosus* (Miniopterus, Miniopteridae) and *Myotis daubentonii* (Myotis, Vespertilioninae), both species belong to the suborder Microchiroptera of the order Chiroptera [18]. The isolation of YOKVs from microbats on two separate occasions suggests that microbats may be suitable hosts for YOKV, although the YOKV replicates poorly in the fruit bat [9].

Bats are mammals that can fly and are characterized by a large number of species and wide distribution. Many studies have shown that bats can carry and spread a variety of pathogens that can cause human and animal disease [19], including SARS [20], Ebola virus [21], and influenza virus [22]. After years of monitoring, a large number of recombinant coronaviruses were discovered in bats, further suggesting that bats may be the host animal for SARS [23]. Given that Oita-36 and XYBX1332 were isolated from the serum of bats, it has been suggested that YOKV can exist in bats, may be occasionally in the long term. How is the virus transmitted to bats? In recent years, Kaeng Khoi virus [10], *Orthoreovirus* [24], *Bunyavirus* [25], and *Rhabdovirus* [26] have been isolated from bat flies (*Eucampsipoda sundaica*), an ectozoon that resides on bats. Whether a cycle including bat flies or other blood-sucking insects (vectors) and bats (hosts) present in the wild to maintain and spread YOKV will require further studies.

## Conclusion

The isolation of YOKV (XYBX1332) from inland China thousands of kilometers from Yokosuka, Japan, suggests that the geographical distribution of YOKV is not limited to the islands of Japan and that it can also exist in the inland areas of Asia. However, there are large differences between the Chinese and Japanese YOKV strains in viral genome.
